# Ultra-High-Performance Liquid Chromatography-Tandem Mass Spectrometry Assay for Quantifying Fentanyl and 22 Analogs and Metabolites in Whole Blood, Urine, and Hair

**DOI:** 10.3389/fchem.2019.00184

**Published:** 2019-04-02

**Authors:** Francesco Paolo Busardò, Jeremy Carlier, Raffaele Giorgetti, Adriano Tagliabracci, Roberta Pacifici, Massimo Gottardi, Simona Pichini

**Affiliations:** ^1^Section of Legal Medicine, Università Politecnica Delle Marche, Ancona, Italy; ^2^Unit of Forensic Toxicology, Università la Sapienza, Rome, Italy; ^3^National Centre on Addiction and Doping, Istituto Superiore di Sanità, Rome, Italy; ^4^Comedical S.r.l., Trento, Italy

**Keywords:** fentanyl, fentanyl analogs, UHPLC-MS/MS, blood, urine, hair

## Abstract

Recently, synthetic opioid-related overdose fatalities, led by illicitly manufactured fentanyl and analogs, increased at an alarming rate, posing a global public health threat. New synthetic fentanyl analogs have been constantly emerging onto the drug marked for the last few years, to circumvent the laws and avoid analytical detection. Analytical methods need to be regularly updated to keep up with the new trends. In this study, we aimed to develop a new method for detecting the newest fentanyl analogs with a high sensitivity, in whole blood, urine, and hair. The method is intended to provide to clinical and forensic toxicologists a tool for documenting consumption. We developed a comprehensive ultra-high-performance liquid chromatography-tandem mass spectrometry method for quantifying fentanyl and 22 analogs and metabolites. Urine samples were simply diluted before injection; a liquid-liquid extraction was performed for blood testing; and a solid phase extraction was performed in hair. The chromatographic separation was short (8 min). The method was validated with a high sensitivity; limits of quantifications ranged from 2 to 6 ng/L in blood and urine, and from 11 to 21 pg/g in hair. The suitability of the method was tested with 42 postmortem blood, urine, or hair specimens from 27 fatalities in which fentanyl analogs were involved. Average blood concentrations (±SD) were 7.84 ± 7.21 and 30.0 ± 18.0 μg/L for cyclopropylfentanyl and cyclopropyl norfentanyl, respectively (*n* = 8), 4.08 ± 2.30 μg/L for methoxyacetylfentanyl, (*n* = 4), 40.2 ± 38.6 and 44.5 ± 21.1 μg/L for acetylfentanyl and acetyl norfentanyl, respectively (*n* = 3), 33.7 and 7.17 μg/L for fentanyl and norfentanyl, respectively (*n* = 1), 3.60 and 0.90 μg/L for furanylfentanyl and furanyl norfentanyl, respectively (*n* = 1), 0.67 μg/L for sufentanil (*n* = 1), and 3.13 ± 2.37 μg/L for 4-ANPP (*n* = 9). Average urine concentrations were 47.7 ± 39.3 and 417 ± 296 μg/L for cyclopropylfentanyl and cyclopropyl norfentanyl, respectively (*n* = 11), 995 ± 908 μg/L for methoxyacetylfentanyl, (*n* = 3), 1,874 ± 1,710 and 6,582 ± 3,252 μg/L for acetylfentanyl and acetyl norfentanyl, respectively (*n* = 5), 146 ± 318 and 300 ± 710 μg/L for fentanyl (*n* = 5) and norfentanyl (*n* = 6), respectively, 84.0 and 23.0 μg/L for furanylfentanyl and furanyl norfentanyl, respectively (*n* = 1), and 50.5 ± 50.9 μg/L for 4-ANPP (*n* = 10). Average hair concentrations were 2,670 ± 184 and 82.1 ± 94.7 ng/g for fentanyl and norfentanyl, respectively (*n* = 2), and 10.8 ± 0.57 ng/g for 4-ANPP (*n* = 2).

## Introduction

Fentanyl is a μ-opioid receptor agonist with strong anesthetic and analgesic properties, with a 50- to 100-fold higher potency than that of morphine. It has been used as a medication for pain management since the 1960s and has been among the most prescribed opioids for the last 3 decades (De Priest et al., 2018). Fentanyl analogs with similar or higher potency, such as sufentanil, alfentanil, and carfentanil, have been subsequently synthesized and used in anesthesia and research (Meert et al., 1988). Since they were first introduced onto the pharmaceutical market, fentanyl and analogs have been misused in place of heroin due to cheaper cost, causing numerous cases of overdose and deaths by respiratory depression, cardiac arrest, or severe anaphylactic reaction (Pichini et al., 2018). Fentanyl and analogs are controlled under Schedule I of the 1961 UN Single convention on narcotic drugs since 1964 (UN, 1961). Following the recent trend of the new psychoactive substances (NPS), new fentanyl analogs started emerging onto the drug market to circumvent the laws and avoid analytical detection (EMCDDA, 2018b). The first substances appeared on the European drug market in 2012 and mostly come from illicit laboratories based in China.

Recently, new synthetic opioids, and more specifically fentanyl and analogs, have been causing a significant spike in intoxications, posing a global public health threat (Prekupec et al., 2017). Illicitly manufactured fentanyl and analogs have been responsible for several thousands of fatalities in the past 3 years (Daniulaityte et al., 2017; O'Donnell et al., 2017; Pichini et al., 2018) and the number of overdose cases is increasing at an alarming rate (Peterson et al., 2016; Rudd et al., 2016; Pichini et al., 2017; EMCDDA, 2018b; Scholl et al., 2018). In USA, new synthetic opioids impacted the demographics of opioid-related overdoses, traditionally associated with heroin and methadone (Rudd et al., 2016; Scholl et al., 2018). In 2017, synthetic opioids were involved in 59.8% of opioid-involved overdose fatalities in the USA (28,466 fatalities involving synthetic opioids other than methadone), which represents an increase of 45.2% from 2016 to 2017 (Scholl et al., 2018). These fatal overdoses were dominated by illicit fentanyl and analogs (Daniulaityte et al., 2017; O'Donnell et al., 2017). Recently, new synthetic opioids have also raised concerns in Europe: the number of seizures of fentanyl analogs was multiplied by 4 between 2015 and 2016 (1,200 seizures in 2016), and many cases of fatal intoxications were reported (EMCDDA, 2018b). Illicit fentanyl, acetylfentanyl, furanylfentanyl, and carfentanil are well-established synthetic opioids and still on the rise (Daniulaityte et al., 2017; O'Donnell et al., 2017). More recent substances, such as cyclopropylfentanyl, methoxyacetylfentanyl, and valerylfentanyl are little known, and have been involved in several deaths in Europe (Pichini et al., 2017; EMCDDA, 2018c).

The number of intoxications and fatalities involving fentanyl and analogs might be underestimated, as (1) they are likely taken in combination with another opioid and may be unnoticed (Daniulaityte et al., 2017), (2) confirmatory analytical tests are not always performed for opioid-involved overdose fatalities and fentanyl analogs overdoses are likely thought to be due to fentanyl (Peterson et al., 2016), and (3) the low active concentrations of fentanyl analogs in biological samples are challenging to detect and require specialized highly-sensitive analytical instruments (Concheiro et al., 2018). In addition, analytical methods need to be constantly updated to keep up with the constant emergence of new uncontrolled analogs.

In this study, we developed a new method by ultra-high-performance liquid chromatography-tandem mass spectrometry (UHPLC-MS/MS) to quantify fentanyl and 22 analogs and metabolites in whole blood, urine, and hair. We aimed to provide a fast, simple, and sensitive analytical tool for clinical and forensic toxicologists to document the consumption of the most recent fentanyl analogs described in the scientific literature: acetylfentanyl, acetyl norfentanyl, alfentanil, butyrylfentanyl, butyrylfentanyl carboxy metabolite, butyryl norfentanyl, carfentanil, cyclopropylfentanyl, cyclopropyl norfentanyl, despropionylfentanyl (4-ANPP), despropionyl *para*-fluorofentanyl, fentanyl, furanylfentanyl, furanyl norfentanyl, furanylethyl fentanyl, β-hydroxyfentanyl, β-hydroxythiofentanyl, methoxyacetylfentanyl, methoxyacetyl norfentanyl, norfentanyl, phenylacetyl fentanyl, sufentanil, and valerylfentanyl carboxy metabolite were included. We confirmed the suitability of the method by testing 42 samples from 27 postmortem cases.

## Materials and Methods

### Chemicals and Reagents

Working standards (acetylfentanyl, acetyl norfentanyl, alfentanil, butyrylfentanyl, butyrylfentanyl carboxy metabolite, butyryl norfentanyl, carfentanil, cyclopropylfentanyl, cyclopropyl norfentanyl, 4-ANPP, despropionyl *para*-fluorofentanyl, fentanyl, furanylfentanyl, furanyl norfentanyl, furanylethyl fentanyl, β-hydroxyfentanyl, β-hydroxythiofentanyl, methoxyacetylfentanyl, methoxyacetyl norfentanyl, norfentanyl, phenylacetyl fentanyl, sufentanil, and valerylfentanyl carboxy metabolite) and deuterated internal standards (IS; acetyl norfentanyl-D5 and fentanyl-D5) were purchased from Cayman Chemical (Ann Arbor, MI, USA) and stored at −20°C until use. LC-MS grade water, acetonitrile, methanol, and formic acid and LC grade acetone and dichloromethane were obtained from Sigma-Aldrich® (Milano, Italy). Ammonium acetate buffer was prepared with ≥97% purity ammonium acetate salt (Sigma-Aldrich®) dissolved in LC-MS water. VMA-T M3®, Washing Solution, and Multimatrix Eluent were acquired from Comedical® s.r.l. (Trento, Italy); their composition is not disclosed.

### Calibrators and Quality Control Solutions

Stock solutions of each standard at 10 mg/L were prepared in methanol. Standard stock solution containing all 23 non-deuterated standards was prepared in methanol at 1 mg/L. IS standard stock solution with acetyl norfentanyl-D5 and fentanyl-D5 was prepared in methanol at 1 mg/L. Stock solutions were stored in glass vials at −20°C.

Calibrator working solutions were daily prepared from the standard stock solution in methanol (5 calibrators along the working range). Low, medium, and QC working solutions were daily prepared from the standard stock solution in methanol. IS working solution was daily prepared from the IS stock solution in methanol to reach a concentration of 5 μg/L in urine and blood samples and 5 ng/g in hair samples.

### Human Samples

Blank human blood, urine, and hair were obtained from the laboratory storehouse of blank biological samples. Pools of blank samples were prepared using 20 different post mortem blood, urine, or hair samples from the Section of Legal Medicine (Università Politecnica delle Marche, Ancona, Italy), pre-screened for the presence of any drug of abuse and pharmaceutical. Postmortem blood, urine, and hair specimens from authentic cases of consumption were provided as discarded material by the Institute of Forensic Medicine of Strasbourg (France), and the Department of Medical and Health Sciences, Division of Drug Research of Linköping University (Sweden). Demographics, detection of other drugs, and cause of death were not specified.

### Sample Preparation

Blood samples (100 μL) were fortified with 5 μL IS working solution, 70 μL M3® reagent (acidic aqueous buffer Busardò et al., 2017; Grabenauer et al., 2018), and 500 μL acetone:acetonitrile 8:2 (*v*/*v*) in polypropylene microcentrifuge tubes. Tubes were capped, vortexed for 10 s, and centrifuged at 15,000 g for 5 min. Supernatants were transferred onto conical glass tubes and evaporated to dryness under nitrogen at 45°C. Samples were reconstituted with 1 mL mobile phase A:B 95:5 (*v*/*v*) and centrifuged at 15,000 g for 5 min. Supernatants were transferred into autosampler glass vials, prior to injection onto the chromatographic system.

Urine samples (100 μL) were fortified with 5 μL IS working solution in conical glass tubes and vortexed. After adding 3 mL mobile phase A:B 95:5 (*v*/*v*), tubes were capped, vortexed for 10 s, and centrifuged at 15,000 g for 5 min. Supernatant was transferred into autosampler glass vials, prior to injection onto the chromatographic system.

Hair samples were washed twice with dichloromethane and dried under nitrogen at 45°C. An amount of 25 mg was cut into pieces (<5 mm) in glass tubes and fortified with 5 μL IS working solution. After addition of 500 μL M3® reagent, tubes were capped and incubated at 100°C for 1 h, for complete hair digestion. Tubes were cooled down at room temperature and samples underwent solid phase extraction on 30 mg/1 mL Oasis® PRIME HLB cartridges (Waters®): samples were diluted with 0.5 mL M3® reagent and loaded onto the cartridges; cartridges were then washed with 0.5 mL Comedical® Washing Solution and dried under nitrogen; elution was performed with 0.5 mL Multimatrix Eluent. Eluates were diluted with 9.5 mL water and 1 mL was transferred into autosampler glass vials, prior to injection onto the chromatographic system.

### Instrumentation

UHPLC-MS/MS analysis was performed on a Waters® Xevo® TQ-S micro mass spectrometer (triple quadrupole) equipped with an electrospray ionization source in positive ion mode (ESI+) and interfaced with an ACQUITY UPLC® I-Class (Waters® Milano, Italy). Data were acquired with MassLynx® software version 4.1 (Waters®).

Separation was performed on an ACQUITY UPLC® BEH C_18_ column from Waters® (length: 50 mm, internal diameter: 2.1 mm, particle size: 1.7 μm). Run time was 8 min with a gradient mobile phase composed of 0.1% formic acid in 5 mM ammonium acetate buffer (A) and 0.05% formic acid in acetonitrile (B) at a flow rate of 0.35 mL/min. Initial conditions were 5% B, held for 1 min, increased to 30% B within 3.5 min, increased to 95% B within 0.5 min, held for 0.5 min, returned 5% B within 0.1 min, and then held for 2.4 min. LC flow was directed to waste the first 1.5 min of the separation and after 6 min. Autosampler and column oven temperatures were 10 and 50°C, respectively. The injection volume was 10 μL for blood and urine samples, 1 μL for hair samples.

The mass spectrometer operated in scheduled multiple reaction monitoring (MRM) mode, with two transitions for each analyte and one transition for each IS (Table 1). MS parameter settings were optimized by infusing neat standards individually in methanol and ramping cone voltage and collision energy (Table 1). Scan speed (dwell time) was adjusted in the chromatographic conditions of the analysis to produce 15 to 20 scans per chromatographic peak. ESI+ conditions were optimized as follows: capillary voltage = 0.5 kV, source temperature = 150°C, desolvation temperature = 650°C, cone gas flow rate = 20 L/h, desolvation gas flow rate = 1,200 L/h.

**Table 1:** Mass spectrometry parameters for analytes and internal standards. Scan speed (dwell time) and detection windows were adjusted accordingly.

**Compound**	**IS**	**Cone voltage (V)**	**Q1 mass (m/z)**	**Quantification transition**		**Confirmation transition**		**RT (min)**
				**Q3 mass (m/z)**	**CE (eV)**	**Q3 mass (m/z)**	**CE (eV)**	
Acetyl norfentanyl-D5 (A-D5)	-	25	224.2	84.0	18	-	-	2.23
Fentanyl-D5 (F-D5)	-	25	342.2	105.2	38	-	-	4.76
Acetylfentanyl	A-D5	25	322.2	188.0	20	105.0	36	4.18
Acetyl norfentanyl	A-D5	25	219.2	84.1	18	55.2	36	2.28
Alfentanil	F-D5	24	417.1	268.1	16	197.1	26	4.80
Butyrylfentanyl	F-D5	30	351.2	105.0	40	188.1	22	5.11
Butyrylfentanyl carboxy metabolite	F-D5	25	381.2	105.0	42	188.0	30	4.05
Butyryl norfentanyl	A-D5	25	247.1	84.2	20	55.3	36	3.80
Carfentanil	F-D5	22	395.2	113.0	32	335.0	16	5.08
Cyclopropylfentanyl	F-D5	25	349.2	105.0	36	188.1	20	4.97
Cyclopropyl norfentanyl	F-D5	25	245.2	84.1	36	177.1	20	3.41
Despropionylfentanyl (4-ANPP)	F-D5	22	281.2	105.0	32	188.0	14	4.77
Despropionyl *para*-fluorofentanyl	F-D5	15	299.1	105.0	38	188.1	16	4.89
Fentanyl	F-D5	25	337.2	188.2	30	105.2	38	4.75
Furanylfentanyl	F-D5	16	375.1	188.0	18	105.0	38	4.87
Furanyl norfentanyl	A-D5	16	271.0	84.2	18	55.1	38	3.24
Furanylethyl fentanyl	F-D5	25	327.2	95.1	35	178.1	16	4.18
β-Hydroxyfentanyl	F-D5	25	389.2	111.0	38	238.0	16	5.15
β-Hydroxythiofentanyl	F-D5	25	359.2	192.0	22	111.0	38	4.12
Methoxyacetylfentanyl	A-D5	25	353.3	188.1	20	105.0	38	4.07
Methoxyacetyl norfentanyl	A-D5	15	249.0	84.1	14	55.0	38	2.19
Norfentanyl	F-D5	25	233.1	84.3	20	55.3	34	3.10
Phenylacetyl fentanyl	A-D5	46	399.3	105.1	44	188.1	24	5.19
Sufentanil	F-D5	16	387.2	111.0	38	238.1	18	5.15
Valerylfentanyl carboxy metabolite	F-D5	40	395.3	105.3	44	188.1	26	4.17

*CE, collision energy; IS, internal standard; RT, retention time*.

### Method Validation

The method was validated in whole blood, urine, and hair following the most recent criteria for method development and validation in analytical toxicology (Peters et al., 2018; Wille et al., 2018). Working ranges were LOQ−100 μg/L in blood and urine, and LOQ−100 ng/g in hair, for all analytes. Selectivity, linearity, sensitivity (limits of detection and quantification), accuracy, precision, and carryover were calculated using five different daily replicates of calibration points (five points for each calibration curve, including the limit of quantification as the lowest point) and five replicates of QC samples (low QC = 0.01 μg/L, medium QC = 10 μg/L, and high QC = 80 μg/L in blood and urine; low QC = 0.01 ng/g, medium QC = 10 ng/g, and high QC = 80 ng/g in hair) along three subsequent working days, as previously described. Analytical recovery and matrix effect (ME) were determined using the experimental design proposed by Matuszewski et al. (2003): set 1 was composed of 5 replicates of analytes diluted in the mobile phase (low, medium, and high QC concentrations); sets 2 and 3 were composed of 5 replicates of pooled blank samples fortified with analytes after and before extraction, respectively (low, medium, and high QC concentrations); for each analyte and concentration, ME was calculated by dividing mean peak areas of set 2 by set 1, and recovery was calculated by dividing mean peak areas of set 3 by set 2. Dilution integrity was tested for over-the-curve samples with a concentration 10 and 50 times higher than the highest calibrators, with a dilution in mobile phase A:B 95:5 (*v*/*v*) before sample treatment. Calibration points and QC samples were prepared by two different staff members.

## Results

**Figure 1:**
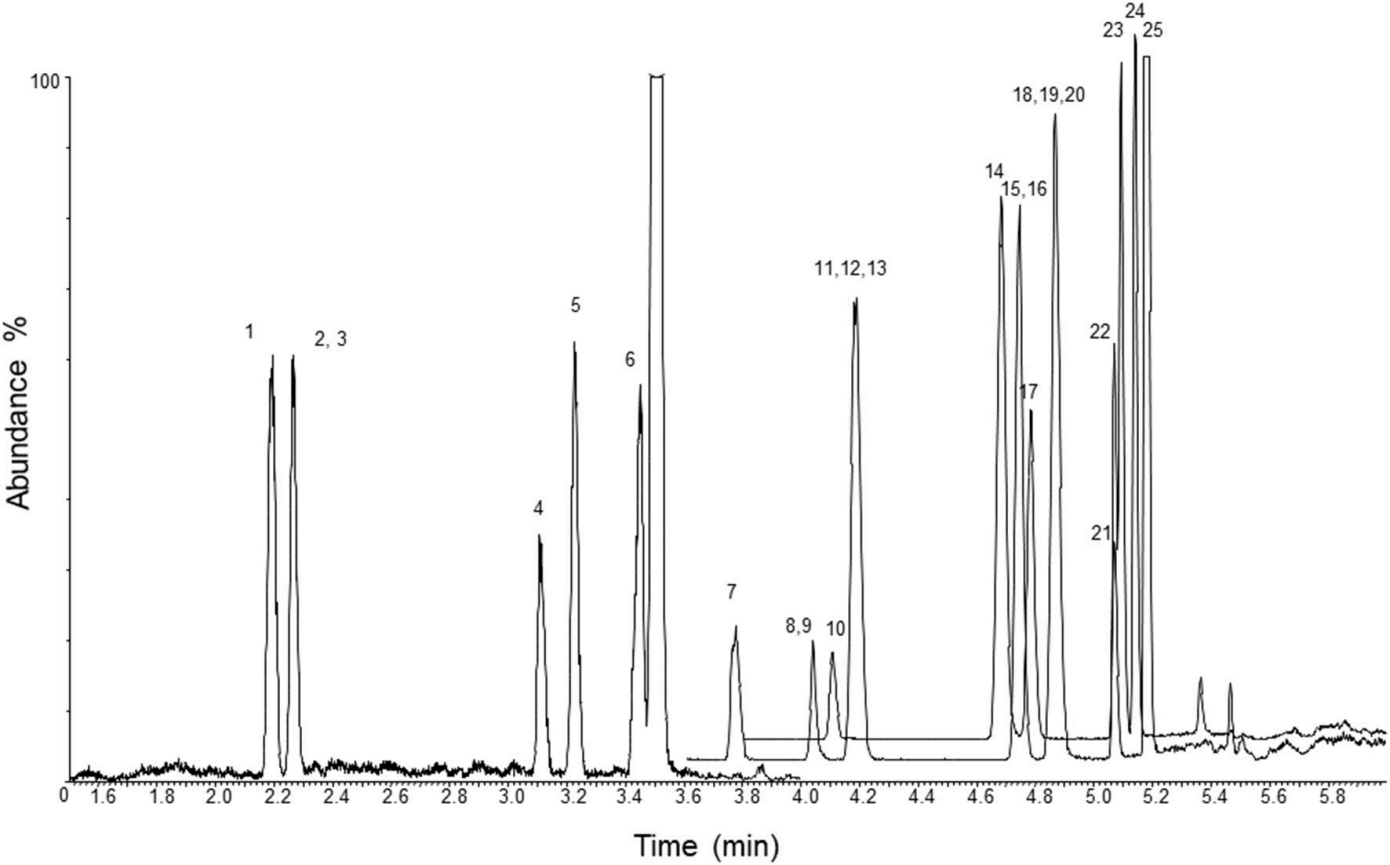
LC-MS/MS chromatogram of a whole blood sample fortified with the analytes at the limit of quantification and their ISs. 1, methoxyacetyl norfentanyl; 2, acetyl norfentanyl; 3, acetyl norfentanyl-D5; 4, norfentanyl; 5, furanyl norfentanyl; 6, cyclopropyl norfentanyl; 7, butyryl norfentanyl; 8, butyrylfentanyl carboxy metabolite; 9, methoxyacetylfentanyl; 10, β-hydroxythiofentanyl; 11, valerylfentanyl carboxy metabolite; 12, furanylethyl fentanyl; 13, acetylfentanyl; 14, fentanyl; 15, fentanyl-D5; 16, despropionylfentanyl (4-ANPP); 17, alfentanil; 18, furanylfentanyl; 19, despropionyl *para*-fluorofentanyl; 20, cyclopropylfentanyl; 21, carfentanil; 22, butyrylfentanyl; 23, sufentanil; 24, β-hydroxyfentanyl; 25, phenylacetyl fentanyl.

**Figure 2:**
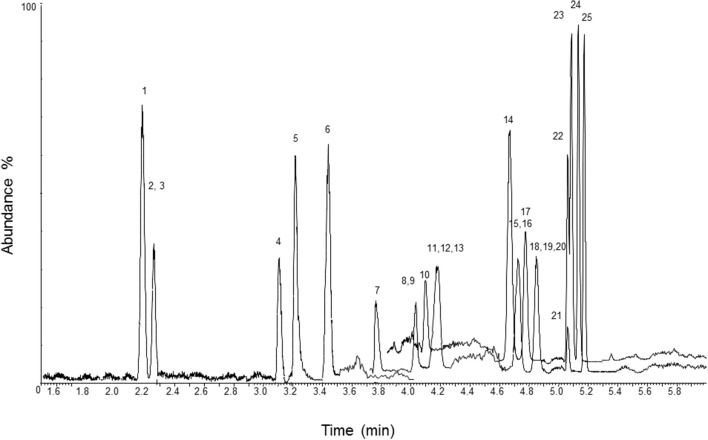
LC-MS/MS chromatogram of a urine sample fortified with the analytes at the limit of quantification and their ISs. 1, methoxyacetyl norfentanyl; 2, acetyl norfentanyl; 3, acetyl norfentanyl-D5; 4, norfentanyl; 5, furanyl norfentanyl; 6, cyclopropyl norfentanyl; 7, butyryl norfentanyl; 8, butyrylfentanyl carboxy metabolite; 9, methoxyacetylfentanyl; 10, β-hydroxythiofentanyl; 11, valerylfentanyl carboxy metabolite; 12, furanylethyl fentanyl; 13, acetylfentanyl; 14, fentanyl; 15, fentanyl-D5; 16, despropionylfentanyl (4-ANPP); 17, alfentanil; 18, furanylfentanyl; 19, despropionyl *para*-fluorofentanyl; 20, cyclopropylfentanyl; 21, carfentanil; 22, butyrylfentanyl; 23, sufentanil; 24, β-hydroxyfentanyl; 25, phenylacetyl fentanyl.

**Figure 3:**
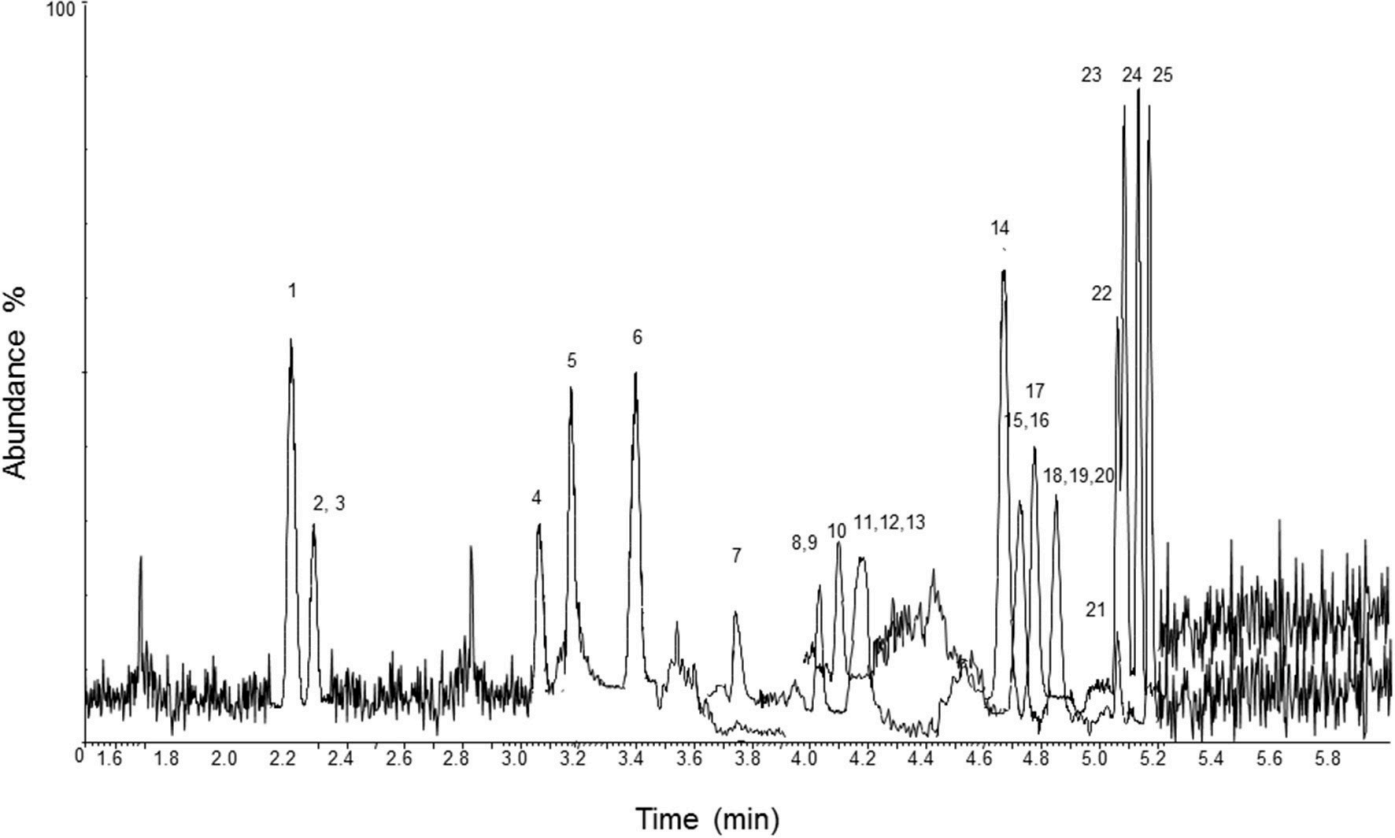
LC-MS/MS chromatogram of a hair sample fortified with the analytes at the limit of quantification and their ISs. 1, methoxyacetyl norfentanyl; 2, acetyl norfentanyl; 3, acetyl norfentanyl-D5; 4, norfentanyl; 5, furanyl norfentanyl; 6, cyclopropyl norfentanyl; 7, butyryl norfentanyl; 8, butyrylfentanyl carboxy metabolite; 9, methoxyacetylfentanyl; 10, β-hydroxythiofentanyl; 11, valerylfentanyl carboxy metabolite; 12, furanylethyl fentanyl; 13, acetylfentanyl; 14, fentanyl; 15, fentanyl-D5; 16, despropionylfentanyl (4-ANPP); 17, alfentanil; 18, furanylfentanyl; 19, despropionyl *para*-fluorofentanyl; 20, cyclopropylfentanyl; 21, carfentanil; 22, butyrylfentanyl; 23, sufentanil; 24, β-hydroxyfentanyl; 25, phenylacetyl fentanyl.

A chromatogram of blood, urine, and hair samples fortified with the analytes at the LOQ and their ISs is displayed in Figures 1–3. Validation parameters in human whole blood, urine, and hair are reported in Tables 2–4, respectively. No additional peaks due to endogenous substances that could have interfered with the detection of the analytes and ISs were observed. Limits of detection (LOD) ranged from 0.7 to 2 ng/L in blood and urine, and from 3 to 7 pg/g in hair. Limits of quantification (LOQ) ranged from 2 to 6 ng/L in blood and urine, and from 11 to 21 pg/g in hair; accuracy and precision were within ±20% of target at the LOQ. Recoveries ranged from 70.7 to 95.7% in blood, from 74.9 to 97.3% in urine, and from 74.3 to 95.7% in hair. No significant ion suppression (<10% analytical signal suppression with CV between 0.2 and 0.9%) due to ME occurred during chromatographic runs. All QCs quantified within ±15% of target for accuracy and intra- and inter-assay precision. Sample contamination by carryover was not observed for any of the 23 analytes. Diluted over-the-curve samples well-fitted into the calibration curves with precision and accuracy (within ±15% of target).

**Table 2:** Validation parameters for fentanyl and analogs in whole blood.

**Compound**	**Determination coefficient (*r*^**2**^)**	**LOD (μg/L)**	**LOQ (μg/L)**	**Accuracy (% error)**			**Intra-assay precision (% CV)**			**Inter-assay precision (% CV)**			**Recovery (%) *n* = 15**	**Matrix effect (%) *n* = 15**
				**Low *n* = 15**	**Mid *n* = 15**	**High *n* = 15**	**Low *n* = 15**	**Mid *n* = 15**	**High *n* = 15**	**Low *n* = 15**	**Mid *n* = 15**	**High *n* = 15**		
Acetylfentanyl	0.997 ± 0.004	0.001	0.003	12.2	9.6	9.7	12.9	11.2	10.7	11.9	9.2	9.6	85.2	91.1
Acetyl norfentanyl	0.998 ± 0.002	0.001	0.004	13.1	8.1	7.5	12.8	7.0	7.4	12.3	5.8	9.5	91.3	92.3
Alfentanil	0.997 ± 0.002	0.001	0.003	10.5	7.6	9.5	10.9	7.2	10.8	11.4	10.4	11.5	88.3	90.7
Butyrylfentanyl	0.994 ± 0.003	0.001	0.004	11.2	7.8	8.2	10.9	10.8	8.9	11.6	11.1	8.8	90.6	92.2
Butyrylfentanyl carboxy metabolite	0.998 ± 0.006	0.002	0.005	12.5	12.8	7.4	12.2	9.4	10.9	12.9	9.1	9.2	86.9	90.8
Butyryl norfentanyl	0.999 ± 0.001	0.001	0.003	12.6	5.5	3.8	10.4	8.1	9.8	10.7	8.9	10.1	90.7	91.1
Carfentanil	0.998 ± 0.002	0.002	0.005	13.0	8.3	5.9	11.7	11.5	10.9	12.2	8.2	10.9	90.3	90.5
Cyclopropylfentanyl	0.997 ± 0.002	0.001	0.004	13.6	9.2	10.2	12.1	7.3	8.6	12.8	9.3	11.5	93.3	91.3
Cyclopropyl norfentanyl	0.996 ± 0.005	0.001	0.004	10.1	5.2	8.8	10.4	10.0	7.9	12.3	9.4	8.7	95.7	90.8
Despropionylfentanyl (4-ANPP)	0.996 ± 0.005	0.001	0.003	10.6	7.7	7.2	11.5	9.8	9.3	13.4	9.5	10.9	70.7	91.2
Despropionyl *para*-fluorofentanyl	0.996 ± 0.003	0.002	0.005	11.5	8.3	6.8	12.3	4.4	8.0	12.9	9.0	73.0	87.6	91.7
Fentanyl	0.998 ± 0.001	0.001	0.003	13.9	9.2	8.7	12.6	5.7	8.3	12.8	7.1	7.8	92.1	90.9
Furanylfentanyl	0.996 ± 0.002	0.001	0.005	10.7	8.7	8.2	13.5	10.8	9.6	12.5	9.3	10.2	74.7	91.5
Furanyl norfentanyl	0.992 ± 0.004	0.001	0.004	12.6	8.5	5.0	14.2	11.0	3.5	11.5	13.2	9.8	95.2	90.4
Furanylethyl fentanyl	0.991 ± 0.003	0.001	0.004	11.8	5.9	7.7	14.0	10.5	9.4	11.7	11.5	8.5	90.9	90.3
β-Hydroxyfentanyl	0.990 ± 0.002	0.002	0.005	11.2	11.9	10.1	12.1	11.2	13.6	11.9	8.9	7.5	88.5	90.4
β-Hydroxythiofentanyl	0.994 ± 0.004	0.002	0.006	13.6	13.1	10.2	12.3	9.4	8.3	12.7	10.2	11.7	90.2	90.6
Methoxyacetylfentanyl	0.997 ± 0.002	0.002	0.005	11.8	12.3	7.4	11.9	9.7	5.4	12.6	10.0	9.1	80.3	90.5
Methoxyacetyl norfentanyl	0.996 ± 0.002	0.001	0.003	12.1	12.3	7.4	12.6	9.7	5.4	12.8	10.0	9.1	80.3	90.3
Norfentanyl	0.995 ± 0.003	0.0007	0.002	12.5	7.5	6.3	12.8	12.6	8.8	13.2	13.4	11.1	85.8	90.6
Phenylacetyl fentanyl	0.990 ± 0.004	0.0007	0.002	10.1	7.2	6.8	11.9	9.0	7.9	11.9	9.4	7.7	90.7	91.1
Sufentanil	0.995 ± 0.003	0.001	0.003	12.4	5.6	8.9	12.6	9.3	7.1	12.4	10.6	11.7	88.4	91.0
Valerylfentanyl carboxy metabolite	0.997 ± 0.002	0.002	0.005	12.7	9.2	10.3	13.2	7.2	8.6	11.2	9.3	11.5	93.3	90.8

Low, medium, and high quality control working solutions contained all standards at 0.01, 10, and 80 μg/L, respectively.

Analytical recovery and matrix effect are displayed as an average of low, medium, and high quality control concentrations values.

*CV, coefficient of variation; LOD, limit of detection; LOQ, limit of quantification*.

**Table 3:** Validation parameters for fentanyl and analogs in urine.

**Compound**	**Determination coefficient (*r*^**2**^)**	**LOD (μg/L)**	**LOQ (μg/L)**	**Accuracy (% error)**			**Intra-assay precision (% CV)**			**Inter-assay precision (% CV)**			**Recovery (%) *n* = 15**	**Matrix effect (%) *n* = 15**
				**Low *n* = 15**	**Mid *n* = 15**	**High *n* = 15**	**Low *n* = 15**	**Mid *n* = 15**	**High *n* = 15**	**Low *n* = 15**	**Mid *n* = 15**	**High *n* = 15**		
Acetylfentanyl	0.998 ± 0.006	0.001	0.003	10.2	11.6	10.7	9.9	8.0	10.7	12.9	9.2	7.6	86.2	90.1
Acetyl norfentanyl	0.997 ± 0.004	0.001	0.004	13.2	9.1	7.5	11.2	6.0	6.4	12.3	10.8	9.5	95.3	92.0
Alfentanil	0.998 ± 0.010	0.001	0.003	10.1	7.6	7.5	11.8	11.2	9.8	10.9	11.4	9.5	80.3	90.2
Butyrylfentanyl	0.991 ± 0.001	0.001	0.004	10.7	7.8	9.2	11.8	8.8	8.9	11.0	11.1	13.8	90.6	90.2
Butyrylfentanyl carboxy metabolite	0.993 ± 0.002	0.002	0.005	11.4	10.8	11.4	11.7	12.4	11.9	11.3	7.1	8.2	86.9	90.7
Butyryl norfentanyl	0.998 ± 0.002	0.001	0.003	10.2	8.5	4.8	10.9	11.1	8.8	12.1	10.9	8.1	88.7	91.3
Carfentanil	0.995 ± 0.004	0.002	0.005	10.9	11.3	8.9	11.7	10.5	7.9	12.2	10.2	9.9	90.3	90.2
Cyclopropylfentanyl	0.997 ± 0.002	0.001	0.004	12.4	9.2	10.2	12.2	7.3	8.6	12.4	9.3	11.5	93.3	91.1
Cyclopropyl norfentanyl	0.996 ± 0.005	0.001	0.004	10.2	5.2	8.8	11.0	10.0	7.9	12.8	9.4	8.7	95.7	90.4
Despropionylfentanyl (4-ANPP)	0.994 ± 0.005	0.001	0.003	10.7	7.7	5.2	11.9	9.8	11.3	10.6	7.5	4.9	91.7	90.2
Despropionyl *para*-fluorofentanyl	0.994 ± 0.001	0.002	0.005	11.8	9.8	7.8	10.5	4.4	6.0	12.3	3.0	4.3	90.6	91.2
Fentanyl	0.995 ± 0.006	0.001	0.003	12.7	10.2	12.7	11.2	7.7	9.3	12.5	9.1	8.8	90.1	90.1
Furanylfentanyl	0.994 ± 0.002	0.001	0.003	10.8	8.7	8.2	11.4	10.6	9.8	12.8	10.3	10.2	74.9	90.5
Furanyl norfentanyl	0.995 ± 0.005	0.001	0.004	11.9	9.5	7.0	12.4	11.0	8.5	13.9	14.2	9.8	93.2	90.7
Furanylethyl fentanyl	0.997 ± 0.005	0.001	0.004	12.1	5.9	6.7	12.3	10.5	11.4	10.4	8.5	7.5	89.9	90.3
β-Hydroxyfentanyl	0.994 ± 0.003	0.002	0.005	10.4	7.9	8.1	12.3	8.2	11.6	10.9	8.9	7.5	88.5	90.6
β-Hydroxythiofentanyl	0.995 ± 0.003	0.002	0.006	11.2	12.1	7.2	13.1	11.4	7.3	11.7	5.2	5.7	85.2	90.1
Methoxyacetylfentanyl	0.997 ± 0.002	0.002	0.005	11.7	12.3	7.4	11.3	9.7	5.4	12.2	10.0	9.1	80.3	90.3
Methoxyacetyl norfentanyl	0.994 ± 0.003	0.001	0.003	12.3	12.3	10.4	12.7	10.7	11.4	14.0	7.0	9.1	80.3	90.2
Norfentanyl	0.995 ± 0.003	0.0007	0.002	12.4	7.5	6.3	12.5	12.6	8.8	10.8	13.4	11.1	85.8	90.1
Phenylacetyl fentanyl	0.993 ± 0.002	0.0007	0.002	10.3	8.2	9.8	12.6	7.0	11.9	10.9	8.4	9.7	91.7	90.4
Sufentanil	0.994 ± 0.002	0.001	0.003	10.7	5.6	4.9	12.1	11.3	8.1	10.5	11.6	12.7	87.4	91.1
Valerylfentanyl carboxy metabolite	0.996 ± 0.001	0.002	0.005	13.2	11.2	8.3	11.2	11.2	9.6	11.6	8.3	7.5	97.3	90.3

Low, medium, and high quality control working solutions contained all standards at 0.01, 10, and 80 μg/L, respectively.

Analytical recovery and matrix effect are displayed as an average of low, medium, and high quality control concentrations values.

*CV, coefficient of variation; LOD, limit of detection; LOQ, limit of quantification*.

**Table 4:** Validation parameters for fentanyl and analogs in hair.

**Compound**	**Determination coefficient (*r*^**2**^)**	**LOD (ng/g)**	**LOQ (ng/g)**	**Accuracy (% error)**			**Intra-assay precision (% CV)**			**Inter-assay precision (% CV)**			**Recovery (%) *n* = 15**	**Matrix effect (%) *n* = 15**
				**Low *n* = 15**	**Mid *n* = 15**	**High *n* = 15**	**Low *n* = 15**	**Mid *n* = 15**	**High *n* = 15**	**Low *n* = 15**	**Mid *n* = 15**	**High *n* = 15**		
Acetylfentanyl	0.992 ± 0.008	0.004	0.012	12.2	8.6	9.7	13.6	11.0	9.7	13.9	10.2	8.6	81.2	90.6
Acetyl norfentanyl	0.994 ± 0.002	0.003	0.011	11.2	7.1	6.5	12.8	5.0	5.4	13.2	6.8	8.5	92.3	91.2
Alfentanil	0.991 ± 0.010	0.005	0.017	10.3	7.6	6.5	10.8	8.2	7.8	12.1	9.4	10.5	84.3	90.4
Butyrylfentanyl	0.995 ± 0.004	0.005	0.015	10.2	6.8	6.2	10.6	7.8	6.9	11.8	8.1	7.8	89.6	90.1
Butyrylfentanyl carboxy metabolite	0.992 ± 0.001	0.006	0.02	10.7	7.8	6.4	13.5	10.4	8.9	13.9	8.1	7.2	76.9	90.9
Butyryl norfentanyl	0.995 ± 0.001	0.006	0.018	11.2	3.5	2.8	11.8	6.1	7.8	12.5	7.9	9.1	89.7	91.1
Carfentanil	0.993 ± 0.001	0.006	0.019	10.7	5.3	4.9	12.7	8.5	6.9	12.9	9.2	8.9	88.3	90.8
Cyclopropylfentanyl	0.997 ± 0.002	0.006	0.019	12.0	9.2	10.2	10.2	7.3	8.6	7.8	9.3	11.5	93.3	91.0
Cyclopropyl norfentanyl	0.996 ± 0.005	0.006	0.018	10.2	5.2	8.8	10.4	10.0	7.9	12.8	9.4	8.7	95.7	90.3
Despropionylfentanyl (4-ANPP)	0.991 ± 0.002	0.006	0.018	12.2	5.7	5.2	13.9	11.8	10.3	14.2	10.5	8.9	79.7	90.1
Despropionyl *para*-fluorofentanyl	0.997 ± 0.002	0.005	0.015	13.4	9.3	5.8	13.9	3.4	5.0	14.3	5.0	6.3	88.6	91.1
Fentanyl	0.992 ± 0.004	0.003	0.013	12.5	8.2	6.7	10.2	4.7	4.3	11.1	6.1	5.8	89.1	90.7
Furanylfentanyl	0.994 ± 0.002	0.005	0.016	10.5	8.7	8.2	11.5	11.6	9.9	11.8	10.4	10.1	78.9	90.2
Furanyl norfentanyl	0.998 ± 0.004	0.003	0.012	10.7	6.5	6.0	13.6	10.0	4.5	13.0	11.2	10.8	91.2	90.1
Furanylethyl fentanyl	0.994 ± 0.005	0.003	0.014	12.9	4.9	5.7	13.6	9.5	8.4	13.9	10.5	9.5	87.9	90.4
β-Hydroxyfentanyl	0.996 ± 0.002	0.005	0.014	12.3	8.9	9.1	12.6	8.2	7.6	12.8	7.9	6.5	81.5	90.9
β-Hydroxythiofentanyl	0.994 ± 0.001	0.005	0.014	12.7	10.1	8.2	13.2	10.4	9.3	13.7	9.2	8.7	83.2	90.2
Methoxyacetylfentanyl	0.997 ± 0.002	0.005	0.016	12.0	12.3	7.4	10.4	9.7	5.4	11.9	10.0	9.1	80.3	90.5
Methoxyacetyl norfentanyl	0.997 ± 0.002	0.005	0.016	12.5	10.3	9.4	11.2	8.7	7.4	12.6	9.0	8.1	74.3	90.5
Norfentanyl	0.991 ± 0.002	0.005	0.015	12.4	8.5	8.3	12.5	10.6	9.8	12.8	10.4	10.1	88.8	90.8
Phenylacetyl fentanyl	0.992 ± 0.004	0.005	0.015	11.1	6.2	5.8	10.9	8.0	6.9	12.1	7.4	6.7	92.7	90.5
Sufentanil	0.992 ± 0.002	0.006	0.019	10.5	4.6	3.9	10.9	7.3	6.1	11.2	8.6	9.7	85.4	91.3
Valerylfentanyl carboxy metabolite	0.994 ± 0.001	0.007	0.021	11.4	10.2	9.3	10.9	8.2	7.6	11.4	7.3	6.5	94.3	90.8

Low, medium, and high quality control working solutions contained all standards at 0.01, 10, and 80 ng/g, respectively.

Analytical recovery and matrix effect are displayed as an average of low, medium, and high quality control concentrations values.

*CV, coefficient of variation; LOD, limit of detection; LOQ, limit of quantification*.

Fentanyl analogs' concentrations measured in authentic human specimens are reported in Table 5. Samples from 27 cases tested positive for fentanyls or analogs. Samples from 13 cases tested positive for 4-ANPP; average blood concentration (± standard deviation, SD) was 3.13 ± 2.37 μg/L (*n* = 9); average urine concentration was 50.5 ± 50.9 μg/L (*n* = 10); average hair concentration was 10.8 ± 0.57 ng/g (*n* = 2). Samples from 12 cases tested positive for cyclopropylfentanyl and cyclopropyl norfentanyl; average blood concentrations were 7.84 ± 7.21 and 30.0 ± 18.0 μg/L, respectively (*n* = 8); average urine concentrations were 47.7 ± 39.3 and 417 ± 296 μg/L, respectively (*n* = 11). Samples from 8 cases tested positive for fentanyl and norfentanyl and one case for norfentanyl only; blood concentrations were 33.7 and 7.17 μg/L, respectively (*n* = 1); average urine concentrations were 146 ± 318 and 300 ± 710 μg/L, respectively (*n* = 5 and 6, respectively); average hair concentrations were 2,670 ± 184 and 82.1 ± 94.7 ng/g, respectively (*n* = 2). Samples from 5 cases tested positive for acetylfentanyl and acetyl norfentanyl; average blood concentrations were 40.2 ± 38.6 and 44.5 ± 21.1 μg/L, respectively (*n* = 3); average urine concentrations were 1,874 ± 1,710 and 6,582 ± 3,252 μg/L, respectively (*n* = 5). Samples from 4 cases tested positive for methoxyacetylfentanyl; average blood concentration was 4.08 ± 2.30 μg/L (*n* = 4); average urine concentration was 995 ± 908 μg/L (*n* = 3). One sample tested positive for furanylfentanyl and furanyl norfentanyl; blood concentrations were 3.60 and 0.90 μg/L, respectively; urine concentrations were 84.0 and 23.0 μg/L, respectively. One sample tested positive for sufentanil; blood concentration was 0.67 μg/L.

**Table 5:** Fentanyl and analogs' concentration measured in authentic specimens.

**Sample ID**	**Matrix**	**Cyclopropyl-fentanyl**	**Cyclopropyl norfentanyl**	**Methoxy-acetylfentanyl**	**Furanyl- fentanyl**	**Furanyl norfentanyl**	**Sufentanil**	**Acetyl- fentanyl**	**Acetyl norfentanyl**	**4-ANPP**	**Fentanyl**	**Norfentanyl**
#1	U	11.7	117	–	–	–	–	–	–	–	–	–
#2	B	4.50	5.00	–	–	–	–	–	–	–	–	–
	U	105	239	–	–	–	–	–	–	–	–	–
#3	U	7.37	343	–	–	–	–	–	–	–	–	–
#4	B	3.05	54.2	–	–	–	–	–	–	–	–	–
	U	19.7	381	–	–	–	–	–	–	–	–	–
#5	B	6.60	9.93	–	–	–	–	–	–	–	–	–
	U	81.4	126	–	–	–	–	–	–	–	–	–
#6	B	21.7	42.5	–	–	–	–	–	–	–	–	–
	U	105	896	–	–	–	–	–	–	–	–	–
#7	B	16.2	48.2	–	–	–	–	–	–	–	–	–
	U	38.2	405	–	–	–	–	–	–	–	–	–
#8	B	0.80	22.3	–	–	–	–	–	–	–	–	–
	U	28.3	829	–	–	–	–	–	–	–	–	–
#9	U	85.0	517	–	–	–	–	–	–	–	–	–
#10	B	5.12	21.9	–	–	–	–	–	–	–	–	–
#11	B	4.75	35.7	1.09	–	–	–	–	–	0.21	–	–
	U	41.8	720	173	–	–	–	–	–	5.56	–	–
#12	B	–	–	–	–	–	–	–	–	–	–	–
	U	1.55	16.3	–	–	–	–	–	–	–	–	–
#13	B	–	–	4.43	–	–	–	–	–	3.80	–	–
	U	–	–	843	–	–	–	–	–	39.0	–	–
#14	B	–	–	4.11	–	–	–	–	–	3.57	–	–
	U	–	–	1,970	–	–	–	–	–	27.2	–	–
#15	B	–	–	6.69	–	–	–	–	–	4.57	–	–
	U	–	–	–	–	–	–	–	–	1.58	–	–
#16	B	–	–	–	3.60	0.90	–	–	–	7.69	–	–
	U	–	–	–	84.0	23.0	–	–	–	126	–	–
#17	B	–	–	–	–	–	0.67	–	–	–	–	–
#18	B	–	–	–	–	–	–	84.8	66.7	4.27	–	–
	U	–	–	–	–	–	–	2,800	8,920	117	0.75	10.9
#19	B	–	–	–	–	–	–	16.0	24.8	1.16	–	–
	U	–	–	–	–	–	–	2,870	8,360	69.2	0.88	11.9
#20	B	–	–	–	–	–	–	19.9	41.9	2.51	–	–
	U	–	–	–	–	–	–	61.8	7,730	9.44	–	8.34
#21	B	–	–	–	–	–	–	–	–	0.43	33.7	7.17
#22	U	–	–	–	–	–	–	3,630	6,990	110	1.23	8.19
#23	U	–	–	–	–	–	–	7.13	909	–	–	–
#24	U	–	–	–	–	–	–	–	–	0.16	714	1,750
#25	U	–	–	–	–	–	–	–	–	–	12.0	9.89
#26	H	–	–	–	–	–	–	–	–	11.2	2,800	149
#27	H	–	–	–	–	–	–	–	–	10.4	2,540	15.1

Concentration in blood and urine is indicated in μg/L; Concentration in hair is indicated in ng/g. Concentrations are indicated with three significant figures.

*B, whole blood; U, urine; H, hair*.

## Discussion

### Method

To cope with the emerging threat of the new synthetic opioids, several methods for detecting fentanyl and analogs in biological samples were published. The methods developed to detect new synthetic opioids in biological and non-biological matrices were recently reviewed (Gerace et al., 2018; Marchei et al., 2018). Several methods to quantify fentanyl and analogs in blood, urine, and hair were also published. In 2006, Wang and Bernert, developed a method for quantifying 13 fentanyl analogs in urine. The method consisted in a 21-min LC-MS/MS analysis on a C_18_ column, following an automated solid phase extraction (0.5 mL urine) with C_18_ cartridges. The method was successfully validated for 8 analogs with acceptable accuracy and precision; LODs ranged from 00.003 to 0.07 μg/L and LOQs were 0.01 μg/L; recoveries ranged from 64 to 114% and ME ranged from 11 to 96% (Wang and Bernert, 2006). In 2009, Gergov et al. validated a method for quantifying 9 fentanyl analogs, along 16 other opioids, in whole blood and urine. Samples (1 mL) were prepared with a liquid-liquid extraction in basic conditions, and the extracts were analyzed by LC-MS/MS with a separation on a C_18_ column in 33 min. LODs ranged from 0.01–1 to 0.01–2 μg/L in blood and urine, respectively, and LOQs ranged from 0.03 to 7 in both matrices; no significant ion suppression was measured, except for norfentanyl (-31%) (Gergov et al., 2009). In 2018, Shoff et al. developed a screening for detecting 14 fentanyl analogs, along with 30 other opioid-related compounds, in whole blood and urine (no quantification). Samples (1 mL) were prepared with a solid phase extraction with mixed C_8_-ion exchange cartridges (CLEAN SCREEN® from United Chemical Technologies), and the extracts were analyzed by LC-MS/MS with an 11 min separation on a C_18_ column and a data-dependent acquisition. The method was validated for 10 analogs with insufficient sensitivity for several analytes (LODs ranged from 0.1 to 0.5 μg/L in blood) (Shoff et al., 2017). In 2018, Noble et al. developed another screening for detecting 13 fentanyl analogs in whole blood (no quantification). Sample preparation consisted in a simple protein precipitation in acetonitrile and the extracts were analyzed by high-resolution LC-MS/MS with a 15 min separation on a C_18_ column and a data-independent acquisition. The method was validated with 81–98% recoveries and <34% ME, but with a low sensitivity (LODs ranged from 5 to 10 μg/kg) (Noble et al., 2018). Again in 2018, Fogarty et al. developed a method for quantifying 19 fentanyl analogs in whole blood. Similarly to previously published methods, samples (0.5 mL) were prepared by solid phase extraction on CLEAN SCREEN® cartridges and the extracts were analyzed by LC-MS/MS with a separation on a C_18_ column in 19 min. Except for 2 analogs, the method was validated with a 0.1–100 μg/L dynamic range (LOD were not measured) (Fogarty et al., 2018). Moody et al. published a method for quantifying 19 fentanyl analogs in whole blood, and detecting 17 analogs in urine (no quantification). Sample preparation was a protein precipitation of blood and urine specimens (0.5 mL) in acetonitrile, followed by a solid phase extraction on cation exchange cartridges; the extracts were analyzed by LC-MS/MS with a short 6-min separation on a C_18_ column. In blood, LODs and LOQs ranged from 0.0125–0.25 to 0.05–0.5 μg/L, respectively; recoveries were higher than 56% and ME were <33.4% (Moody et al., 2018). Recently, Strayer et al. validated a method for quantifying 24 fentanyl analogs in whole blood. Samples (1 mL) were prepared with a solid phase extraction on CLEAN SCREEN® cartridges, and the extracts were analyzed by LC-MS/MS with a separation a biphenyl column in 13.5 min. A total of 13 analogs were successfully validated with accurate and precise results; LODs and LOQs ranged from 0.016–0.1 to 0.1–0.5 μg/L, respectively; recoveries ranged from 38–140, 33–96, and 91–97% at a low, medium, and high concentration, respectively; ME ranged from 57.3–117.5, 71.85–125, and 47.72–111.89%, respectively (Strayer et al., 2018). Salomone et al. proposed the first method dedicated to the quantification of fentanyl analogs in hair (10 analogs and 3 other opioids). The method duration was short, with an extraction consisting of a simple incubation of 25 mg hair in methanol at 55°C, and a 6 min LC-MS/MS separation on a C_18_ column. LODs ranged from 0.1 to 0.3 ng/g and LOQs ranged from 0.3 to 0.9 ng/g; recoveries ranged from 71 to 112% and matrix effects ranged from 69 to 111% (Salomone et al., 2018).

We developed and validated an LC-MS/MS method for quantifying fentanyl and 22 analogs and metabolites in human whole blood, urine, and hair. Fentanyl analogs and their metabolites were included in the method depending on the current drug trends, the scientific literature (Feierman and Lasker, 1996; Tateishi et al., 1996; Labroo et al., 1997; Goggin et al., 2017; Watanabe et al., 2017; Concheiro et al., 2018; Kanamori et al., 2018a,b), and their commercial availability. To the best of our knowledge, we report the first method for quantifying cyclopropyl norfentanyl, furanylethyl fentanyl, methoxyacetyl norfentanyl, phenylacetyl fentanyl, and valeryl fentanyl carboxy metabolite in biological samples; despropionyl *para*-fluorofentanyl and β-hydroxyfentanyl in urine; and acetyl norfentanyl, carfentanil, cyclopropylfentanyl, despropionyl *para*-fluorofentanyl, furanyl norfentanyl, β-hydroxyfentanyl, β-hydroxythiofentanyl, and methoxyacetylfentanyl in hair. In addition, this is the most comprehensive method for quantifying fentanyl and analogs in blood, urine, and hair. The capillary voltage was low compared to traditional LC-MS methods (0.5 kV), but higher voltages did not provide a better signal so we opted for a low voltage to limit unwanted side reactions and preserve the source. The same capillary voltage was used in other published methods for quantifying fentanyl and analogs in blood and urine, using Waters® technology (Wang and Bernert, 2006; Fogarty et al., 2018; Moody et al., 2018). Matching deuterated ISs could not be purchased for every analyte, but the method was optimized to avoid any significant matrix effect that could have affected the accuracy and the precision of the results. The analytical recovery of several analytes was affected to a limited extent, which also impacted the LODs and LOQs. However, the sensitivity was higher than that of previously published methods for quantifying fentanyl analogs (Wang and Bernert, 2006; Gergov et al., 2009; Shoff et al., 2017; Fogarty et al., 2018; Moody et al., 2018; Noble et al., 2018; Salomone et al., 2018; Strayer et al., 2018), which is essential for the detection of these compounds, considering their potency and low active concentrations (Concheiro et al., 2018). Although the method duration was short compared to other previously published methods (6–33 min), ME were lower (Wang and Bernert, 2006; Moody et al., 2018; Noble et al., 2018; Salomone et al., 2018; Strayer et al., 2018). Recoveries were similar to other methods (Wang and Bernert, 2006; Moody et al., 2018; Noble et al., 2018; Salomone et al., 2018; Strayer et al., 2018), but the extraction was simpler, as most of the methods used a time-consuming solid phase extraction in blood (Shoff et al., 2017; Fogarty et al., 2018; Moody et al., 2018; Strayer et al., 2018) and urine (Wang and Bernert, 2006; Shoff et al., 2017). The hair extraction (simple incubation in methanol) and the LC-MS/MS duration (6 min) were shorter in the method of Salomone et al. (2018), but our method allows the quantification of more fentanyl analogs with higher sensitivity and lower matrix effects.

### Authentic Specimens

We detected fentanyl and analogs in 42 samples from 27 postmortem cases. To the best of our knowledge, this is the first report of cyclopropyl norfentanyl and furanyl norfentanyl concentrations in blood and urine specimens. Cyclopropylfentanyl was the most prevalent substance (*n* = 12), although the drug has been on the drug market for only 2 years (EMCDDA, 2018a), followed by fentanyl (*n* = 9), acetylfentanyl (*n* = 5), methoxyacetylfentanyl (*n* = 4), furanylfentanyl (*n* = 1), and sufentanil (*n* = 1); the other detected substances were metabolites. Drugs were mostly taken alone, although co-consumption of fentanyl and acetylfentanyl was frequent (*n* = 4). Demographics, co-administration of drugs, and cause of death were not specified. Moreover, fentanyl is subject to extensive postmortem redistribution (Brockbals et al., 2018) and the same may apply to its analogs, which limits the interpretation of our results.

Dealkylation is often a major metabolism pathway of fentanyl analogs, making nor-metabolites good biomarkers of consumption (Concheiro et al., 2018): acetyl norfentanyl and norfentanyl are major metabolites of acetylfentanyl (Watanabe et al., 2017; Kanamori et al., 2018a,b), fentanyl (Feierman and Lasker, 1996; Tateishi et al., 1996; Labroo et al., 1997), respectively, and furanyl norfentanyl is a minor metabolite of furanylfentanyl (Goggin et al., 2017). In our study, acetylfentanyl and fentanyl concentrations were higher than those of their nor-metabolites in blood and urine samples, with a few exceptions, confirming their suitability as biomarkers of consumption. Furanyl fentanyl concentration was higher than that of furanyl norfentanyl in our only case of furanylfentanyl intake (case #16), which is also consistent with the scientific literature on furanylfentanyl metabolism (Goggin et al., 2017; Watanabe et al., 2017) and furanylfentanyl/furanyl norfentanyl detection in authentic whole blood and urine samples (Goggin et al., 2017; Strayer et al., 2018), indicating that furanyl norfentanyl is not a suitable biomarker of furanylfentanyl consumption. Data on cyclopropylfentanyl and methoxyacetylfentanyl metabolism are not currently available, and we report the first method for detecting the two analogs in biological samples. Cyclopropyl norfentanyl concentration was higher than that of cyclopropylfentanyl in every blood and urine sample. In case #12, the two substances were found in urine at a low concentration but were not detected in blood, pointing toward a longer delay between drug use and the time of death/sampling. These results indicate that cyclopropyl norfentanyl may be an efficient biomarkers of cyclopropylfentanyl consumption. On the contrary, methoxyacetyl norfentanyl was not detected when methoxyacetylfentanyl was taken, whether in blood or urine, indicating that methoxyacetyl norfentanyl is not a suitable biomarker of methoxyacetylfentanyl consumption.

4-ANPP was the analyte detected in the highest number of cases (*n* = 13). This is not surprising, considering that 4-ANPP is a metabolite of several fentanyl analogs and a well-known chemical intermediary of fentanyl synthesis by Siegfried method (DEA, 2010). Consequently, 4-ANPP was detected in all cases with fentanyl consumption, except for case #25 (urine). 4-ANPP is a major metabolite of methoxyacetylfentanyl (Mardal et al., 2018) and furanylfentanyl (Watanabe et al., 2017) and a minor metabolite of acetylfentanyl (Watanabe et al., 2017), and was detected in every blood and urine samples when one of the three analogs was taken. 4-ANPP blood, urine, and hair concentrations were lower than those of fentanyl, methoxyacetylfentanyl, acetylfentanyl, and their nor-metabolites when one of the three analogs was taken alone, making it an inefficient and non-specific marker of consumption. However, 4-ANPP blood and urine concentrations were higher than those of furanylfentanyl and furanyl norfentanyl in our only case of furanyl intake (case #16), as described in previous cases (Goggin et al., 2017; Martucci et al., 2018). Consequently, although it is not specific of furanylfentanyl intake, 4-ANPP may be a better marker than furanyl norfentanyl for documenting furanylfentanyl intake.

The concentration of fentanyl and analogs in urine was always higher than that of blood, when the two matrices were available for the same subject: the elimination of fentanyl analogs and their metabolites in urine appears to be significant, making urine a matrix of choice for documenting consumption in clinical or forensic toxicology. As expected, fentanyl concentration was higher than that of norfentanyl and 4-ANPP in hair, as hair usually contains a high parent drug to metabolite ratio (Tzatzarakis et al., 2017).

## Conclusion

We developed the most comprehensive method for quantifying fentanyl analogs and metabolites in human whole blood, urine, and hair, for clinical and forensic applications. The method is simple and fast, allowing automation and high-throughput testing, and was validated in the three matrices with a high sensitivity for all the analytes. This is the first reported method for quantifying cyclopropyl norfentanyl, furanylethyl fentanyl, methoxyacetyl norfentanyl, phenylacetyl fentanyl, and valeryl fentanyl carboxy metabolite in biological samples.

The method was applied to authentic blood, urine, and hair postmortem samples, and 42 tested positive (27 different cases). Cyclopropylfentanyl was the most prevalent analog; acetylfentanyl, fentanyl, furanylfentanyl, methoxyacetylfentanyl, sufentanil, and metabolites were also found. Drugs were mostly taken alone, although co-consumption of fentanyl and acetylfentanyl was frequent. We report the first concentrations of cyclopropyl norfentanyl in blood and urine, and the first concentration of furanyl norfentanyl in blood. Cyclopropyl norfentanyl proved to be a good marker of cyclopropylfentanyl intake. On the contrary, methoxyacetyl norfentanyl was not a suitable marker of methoxyacetylfentanyl consumption. Similarly, furanyl norfentanyl was a poor biomarker of furanylfentanyl intake, and 4-ANPP may be more suitable to document furanylfentanyl consumption, although it is also the metabolite of several other fentanyl analogs.

## Author Contributions

FB, MG, and SP designed the study; JC, RG, AT, and RP approved the design. FB, MG, and SP developed the analytical method and RG, AT, and RP revised the method and performed the analyses. JC drafted the manuscript and all the other authors contributed to manuscript intellectual content and revision.

### Conflict of Interest Statement

The authors declare that the research was conducted in the absence of any commercial or financial relationships that could be construed as a potential conflict of interest.
